# Optical Filters with Asymmetric Transmittance Depending on the Incident Angle, Produced Using Liquid Crystalline Ink (Louver LC Filters)

**DOI:** 10.3390/ma16165584

**Published:** 2023-08-11

**Authors:** Kohki Takatoh, Mika Kobayashi, Masahiro Ito

**Affiliations:** 1 Department of Electrical Engineering, Faculty of Engineering, Sanyo-Onoda City University, 1-1-1 Daigaku-dori, Sanyo-Onoda 756-0884, Japan; 2 Medical Engineering Course, Department of Medical Course, Faculty of Health and Medical Science, Teikyo Heisei University, 2-51-4 Higashi-ikebukuro, Toshima-ku, Tokyo 170-8445, Japan; masahiro.ito@thu.ac.jp

**Keywords:** liquid crystal, liquid crystalline ink, louver filter, HAN-type LC, asymmetric transmittance dependence, pretilt angle, half-wave plate, optical films

## Abstract

In many situations in everyday life, sunlight levels need to be reduced. Optical filters with asymmetric transmittance dependent on the incident angle would be useful for sunglasses and vehicle or architectural windows, among others. Herein, we realized the production of optical filters, called “louver filters”, comprising HAN-type LC film produced using liquid crystalline ink with dichroic dyes. For the formation of the HAN-type LC film, the liquid crystalline ink was aligned on a rubbed polyimide layer and polymerized by UV irradiation. Two kinds of filters are proposed: one is a filter composed of HAN-type LC film and a polarizer, and the other is composed of two HAN-LC films with a half-wave plate between them. The dependence of the asymmetric transmittance on the incident angle was confirmed for these filters. The dependence changed depending on the pretilt angle of the alignment layers. Photographs taken with the optical filters displayed their effectiveness.

## 1. Introduction

Control of light strength is important for all circumstances in life. In the case of windows for buildings and cars, or in sunglasses, only the light from above should be blocked out; light from the front or from below need not be blocked. For these situations, optical films for which transmittance varies depending on the direction are desirable. However, the optical films produced so far have been optically isotropic, and the transmittance does not vary with angle.

Concerning optical films of which the transmittance varies depending on the incident angle, several kinds of films have been commercialized for peeping prevention of flat panel displays [1,2]. These films possess minute layered structures perpendicular or oblique to the film plane. These films must be used by attaching to the displays. In the case of the applications of these films to windows or glasses, the light scattering occurs to make the films opaque. To prevent the light scattering, the size of the layered structures must be less than the light wavelength. On the other hand, it was reported that by using a liquid crystalline solution containing liquid crystalline monomer and dichroic dye, polarizers can be formed [3]. In this method, the dichroic dye alignment direction was controlled by liquid crystalline materials. The dichroic dye exists homogeneously in the film and light scattering does not occur. Concerning the molecular arrangement of the liquid crystal, not only the azimuth angle but also the polar angle can be controlled. By controlling both the azimuth angle and polar angle of dichroic dye in the film, the optical filters with asymmetric transmittance depending on the incident angle without light scattering could be realized.

The authors proposed optical devices using liquid crystal devices (LCDs) with asymmetric transmittance [4,5]. For these devices, hybrid alignment nematic (HAN) LCDs [6,7,8,9,10,11,12] and LCDs of high pretilt parallel alignment with dichroic dye were used. Combinations of one of these LCDs and a polarizer or two of these LCDs and a half-wave plate could realize effectively asymmetric transmittance dependent on the incident angle. By using LCDs, the properties can be changed by applying an electric voltage. However, for use in the windows of buildings and cars or in sunglasses, optical filters composed of film materials would be more appropriate than LCDs. These HAN-type films could be produced by printing liquid crystalline ink containing liquid crystal monomer and dichroic dye on rubbed polyimide layers.

**Figure 1:**
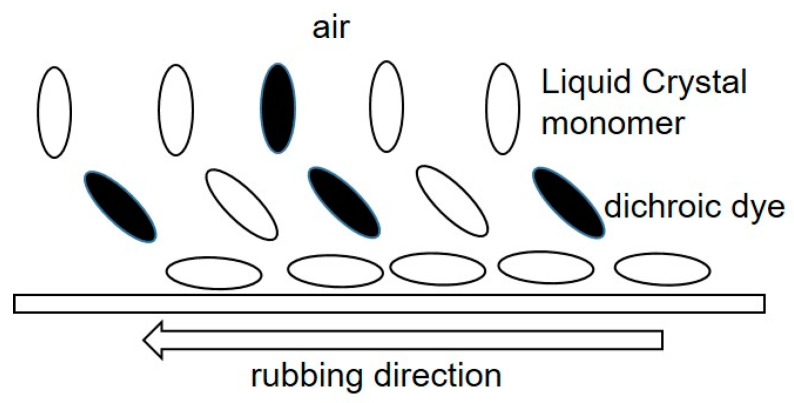
Polymer film possessing an HAN-type LC structure.

It is known that in the interface between liquid crystal (LC) materials and air, the LC molecules tend to align perpendicular to the surface of the interface [13]. Therefore, by placing an LC layer on a rubbed alignment layer for homeotropic alignment, an HAN molecular arrangement can be formed spontaneously. Furthermore, by using LC monomers as LC materials and irradiating them with ultraviolet (UV) light, polymer films possessing an HAN-type structure (Figure 1) can be formed [14,15,16,17,18,19,20,21,22,23].

**Figure 2:**
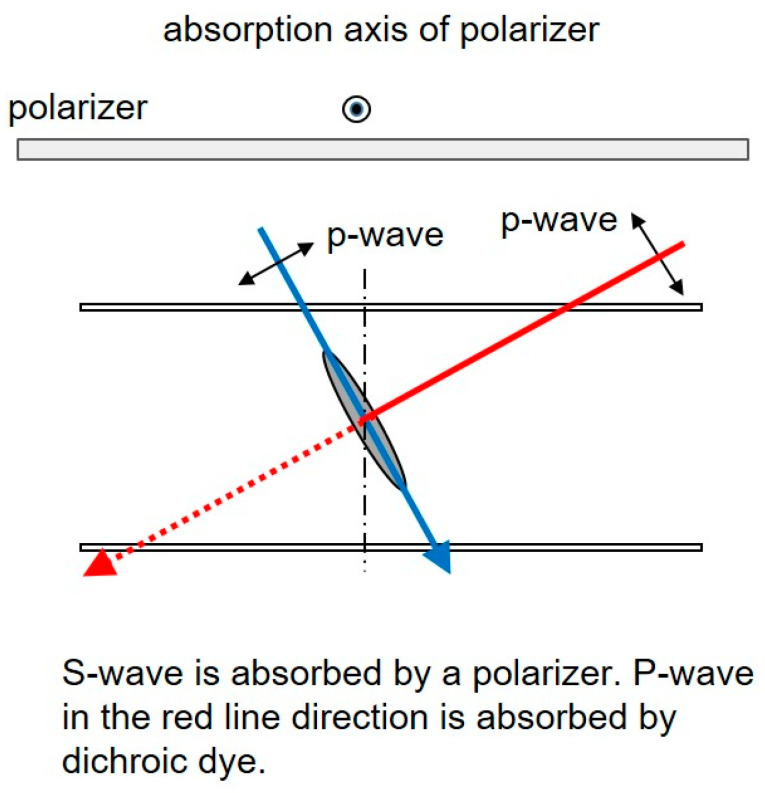
The structure of an optical filter using an HAN-type LC film and a polarizer, and the mechanism for the dependence of the transmittance on the incident angle.

Figure 2 shows the structure of an optical filter using an HAN-type LC film and a polarizer, along with the mechanism for the dependence of the transmittance on the incident angle [4,5]. In the case of the optical filters shown in Figure 2, the s-waves are absorbed by the polarizer. The p-waves in the direction perpendicular to the molecular axis of the dichroic dye are absorbed. On the other hand, the p-waves in the direction parallel to the molecular axis of the dye pass through the film. As a result, dependence of the transmittance on the incident angle becomes possible. When using a polarizer, the transmittance of the filter is less than 50%.

**Figure 3:**
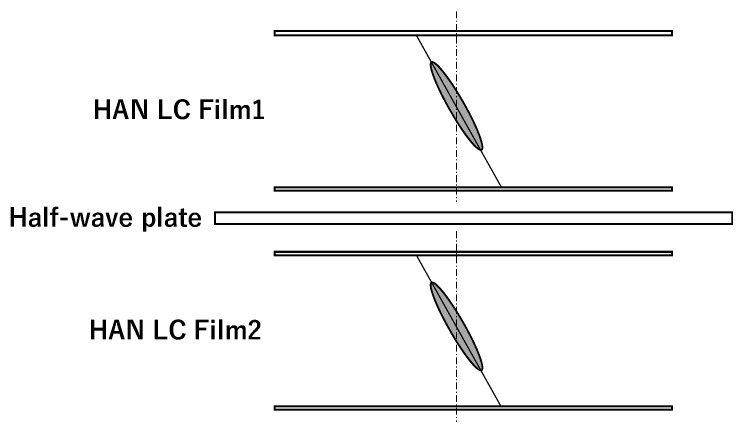
The structure of an optical film using two HAN-type LC films and a half-wave plate between them. The LC alignment directions of the two HAN-type LC films are the same. The angle between the LC alignment directions and the optical axis of the half-wave plate is set at 45 degrees.

In Figure 3, the structure of an optical film made using two HAN-type LC films with a half-wave plate between them is shown. The LC alignment direction or rubbing direction on the alignment film of each HAN-type LC film is the same. The angle between the alignment direction and the optical axis of the half-wave plate is set at 45 degrees. The polar angle of the dye molecules changes through the LC layer. The average polar angle of the molecules is shown [4].

**Figure 4:**
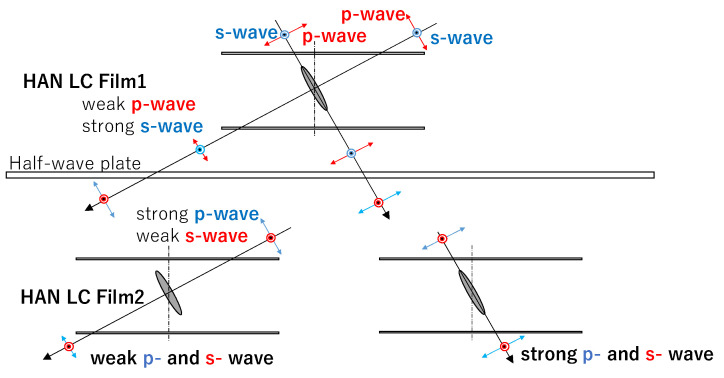
The mechanism realizing the dependence of the transmittance on the incident angle when using the structure shown in Figure 3 [4].

Figure 4 shows the mechanism realizing the dependence of the transmittance on the incident angle when using the structure shown in Figure 3 [4]. The incident light parallel to the dye’s molecular axis passes through the films with little absorption. On the other hand, the p-waves of incident light perpendicular to the dye molecular axis are absorbed by the dye; however, the s-waves of the incident light pass through the film without absorption. By passing through a half-wave plate, p-waves and s-waves are exchanged. In the second LC film, the p-waves exchanged with the s-waves are absorbed by the dichroic dyes. According to this mechanism, the incident light perpendicular to the LC molecules or dichroic dyes is absorbed, while the incident light parallel to them passes through the films with little absorption. In the optical filter shown in Figure 3, no polarizer is used. Thus, the transmittance could be more than 50%.

We call these filters “louver LC filters”. This name refers to the fact that these optical filters are composed of films produced from liquid crystalline ink and to the dependence of the transmittance on the incident angle.

## 2. Materials and Methods

### 2.1. Materials

RMM28B (Merck KGaA, Darmstadt, Germany) [24,25,26] was used as the LC monomer. RMM28B is in a solid state at room temperature. With heating, it enters the LC state at 53 °C. At temperatures higher than 76 °C, it is in both an LC state and a liquid state. With cooling, the LC state is maintained at room temperature. However, after 1 h, it returns to a solid state. RMM28B contains a photoinitiator of polymerization, Irugacure 907 (<10%). For the dichroic dye, NKX-4173 (Hayashibara Co., Ltd., Okayama, Japan) was used.

### 2.2. Preparation of the Liquid Crystalline Ink

Quantities of 200.5 mg of LC monomer RMM28B, 4.0 mg (2 wt% relative to the RMM28B) of dichroic dye NKX-4713, and 328.1 mg of toluene were placed in a sample bottle. The weight proportion of the solid was 38%. The bottle was placed on a hot plate with a magnetic stirrer function. The mixture was stirred at 100 °C for 60 min to obtain a homogeneous black solution.

The state of the solution was observed from 25 to 110 °C using an Olympus BX50 polarizing microscope, (Olympus Co., Tokyo, Japan) a Mettler Toledo FP90, and a hot stage (Mettler-Toledo International, Tokyo, Japan). The solution showed a nematic LC state from 25 to 106 °C. Solutions containing 1, 3, and 5 wt% of dichroic dye NKX-4713 were also prepared.

### 2.3. Preparation of HAN-Type LC Films

#### 2.3.1. Formation of Alignment Layers with a Small Pretilt Angle

A layer of SE-150 polyimide solution (Nissan Chemical Co., Tokyo, Japan) with a pretilt angle of 4 degrees was formed via 3000 rpm rotation spin coating on a glass plate of dimensions 20 mm × 25 mm area and 0.7 mm thickness. The formed layer was heated at 200 °C for 1 h to obtain polyimide film 0.1 μm thick. The surface of the layer was rubbed with a cotton velvet cloth attached to a 40 mm diameter roller rotating at 1000 rpm using a PM-50 rubbing machine (EHC Co., Tokyo, Japan). The distance between the polyimide layer surface and the roller was set to be 0.4 mm shorter than the length of the velvet fiber.

#### 2.3.2. Formation of Alignment Layers with a Large Pretilt Angle

Alignment layers with a large pretilt angle were made by mixing polyimides for homogeneous and for homeotropic alignment, following a method previously reported [27]. For the homogeneous and homeotropic alignment layers, the polyimides PIA-X359-01 (JNC Co., Tokyo, Japan) and PIA-X768-01 were used. By mixing PIA-X359-01 and PIA-X768-01 at ratios of 85:15, 60:40, and 40:60, alignment layers of pretilt angles of 6°, 17°, and 27° could be realized.

#### 2.3.3. HAN-Type Layer Formation by Spin Coating

A solution of LC monomer and dichroic dye (1 wt%) was coated on the surface of an SE-150 alignment layer on a glass plate by rotation at 3000 rpm using a spin coater. Just before use, the solution was heated to 40 °C while stirring with a magnetic stirrer. The layer was heated at 55 °C for 1 min and was irradiated with 28.7 mW/cm^2^ 365 nm UV light for 1 min. The layer thickness was 3.6 μm. For the HAN-type layer produced by spin coating, two areas possessing opposite polar angle directions were observed. The two areas separated at the center of the rotation. By using the same methods, layers containing 2, 3, and 5 wt% dichroic dye were formed. The layer thicknesses were 4.2, 3.2, and 3.8 μm, respectively. HAN-type layers were also prepared on the high-pretilt-angle alignment layers. Optical measurements were carried out in the area in which the polar angle was formed in the same direction as the pretilt angle.

#### 2.3.4. HAN-Type LC Layer Formation Using a Film Applicator

A polyethylene terephthalate (PET) film (95 mm × 95 mm, 0.125 mm thickness) was fixed on a glass plate (100 mm × 100 mm, 0.7 mm thickness) using polyimide tapes. A solution of SE-5291 polyimide (Nissan Chemical Co.) was coated on this film by 3000 rpm spin-coating rotation. The layer was heated at 90 °C for 45 min to obtain a polyimide layer. The surface on the polyimide layer was rubbed as described in Section 2.3.1. The pretilt angle of SE-5291 is 6 degrees [28]. A solution of LC monomer and dichroic dye (5 wt% relative to the LC monomer) was coated on the obtained alignment layer using an SA-201 Baker-type film applicator and a PI-1210 auto film applicator (Tester Sangyo Co., Ltd., Saitama, Japan).

Just before use, the solution was heated to 40 °C. The coating direction was set parallel and opposite to the rubbing direction. The layer thickness and the bar speed of the applicator were set to 20 μm and 50 mm/s, respectively. The applicator was warmed to 55 °C before use. The obtained layer was heated to 55 °C for 1 min. The substrate with the formed LC monomer layer was set in a vacuum using a vacuum vessel with quartz glass (MUVPBQ-150, AITEC SYSTEM Co., Ltd., Kanagawa, Japan). UV light (365 nm, 28.7 mW/cm^2^) was irradiated through the quartz glass for two minutes. The layer thickness was measured using a VK9710/VK9700 laser microscope (KEYENCE Co., Osaka, Japan) and found to be 9 μm.

### 2.4. Combination of Two LC Films

The combination of two LC films and a half-wave plate shown in Figure 3 was formed by using two LC films on glass or PET film substrate. In the case of a glass substrate, an upper glass substrate was placed on another LC film, and the rubbing direction on each substrate was set to be the same. In the case of a PET substrate, each PET substrate was placed on the outside of the two LC films, because PET substrate has the quality of birefringence. The rubbing directions were set to be parallel and opposite. The direction of the half-wave plate extension axis was set at 45 degrees to the rubbing direction. Pure-ace@R-270 polycarbonate film (film thickness 67 μm, TEIJIN Ltd., Tokyo, Japan) was used as the half-wave plate. The retardation of this film was measured and found to be 267 nm using 579 nm light.

### 2.5. Measurement of the Incident Angle Dependence of the Transmittance

The dependence of the transmittance of the optical filters on the incident angle was measured using an RETS-100 optical property measurement system (Otsuka Electronic Co., Osaka, Japan). The transmittance of the single LC films was measured using polarized light (p-waves in Figure 2) with a polarizer. Transmittance without a polarizer was taken as 100%.

**Figure 5:**
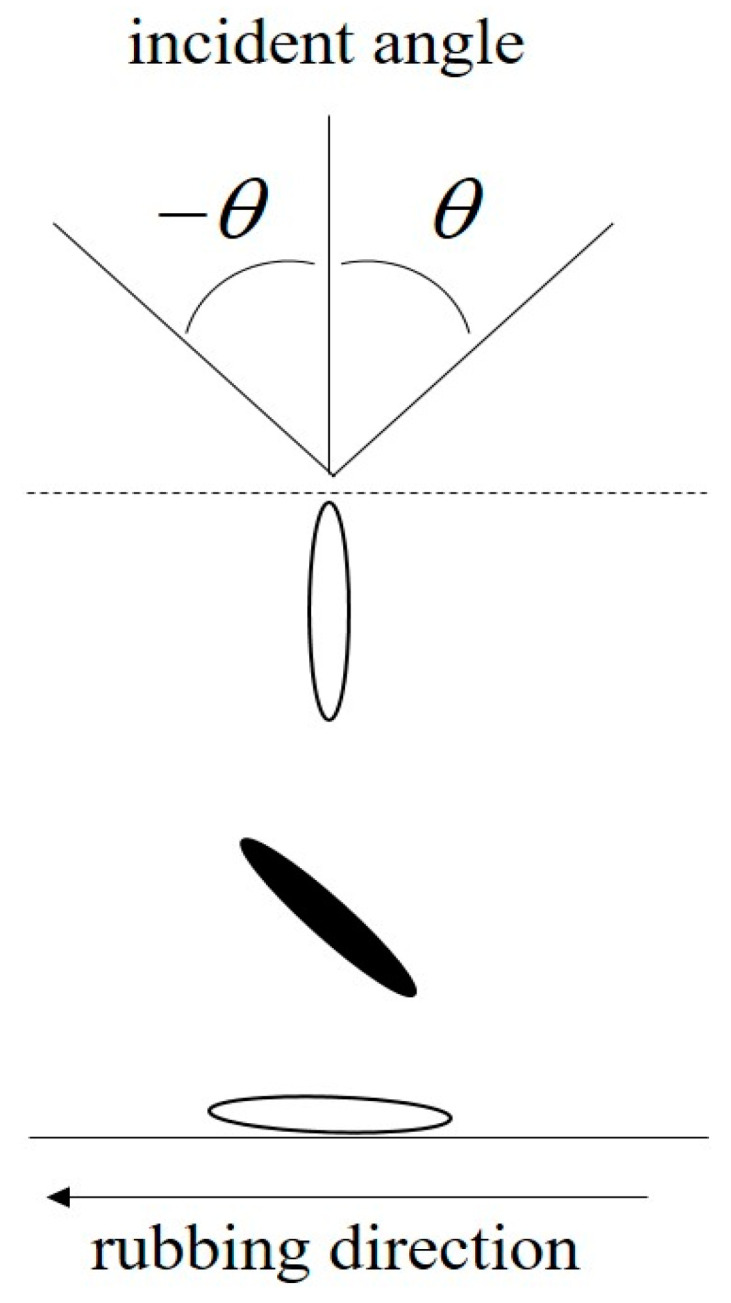
The relationship between the LC monomer polar angle direction and the sign of the incident angle.

The transmittance of the two LC films in Figure 3 was measured using nonpolarized light without a polarizer. The relationship between the sign of the incident angle and the direction of the polar angle of the LC monomer is shown in Figure 5.

### 2.6. Measurement of the Pretilt Angles of the Alignment Layers

In order to measure the alignment layers’ pretilt angles, LC cells with parallel but opposite rubbing directions were prepared. The distance between the alignment layers was set to 20 μm. The pretilt angle was measured using the PAS-301 pretilt-angle measurement system (Elsicon Co., Newark, DE, USA).

## 3. Results and Discussion

### 3.1. HAN-Type LC Layers Formed by Spin Coating

**Figure 6:**
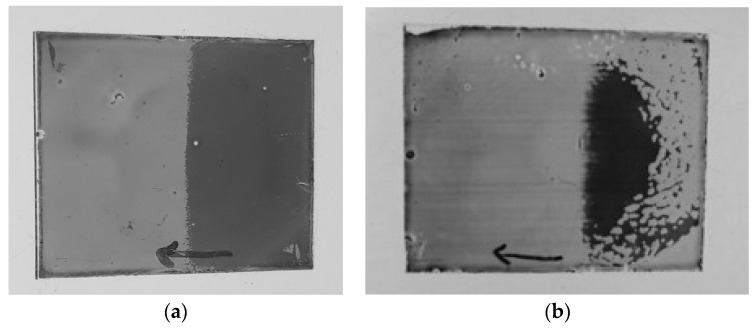
Photographs of LC layers formed by spin coating: (**a**) low-pretilt-angle alignment layer (dark area, irregular region; light area, regular region); (**b**) high-pretilt-angle alignment layer (dark area, irregular region; light area, regular region). The arrow shows the direction of the rubbing process.

Figure 6 shows the photographs from the direction of negative incident angle shown in Figure 5. In the case of spin coating, two kinds of uniform HAN-type LC alignment regions were formed. In particular, in the case of a low-pretilt-angle alignment layer, the boundary between the regions was at the center of the rotation (Figure 6a).

**Figure 7:**
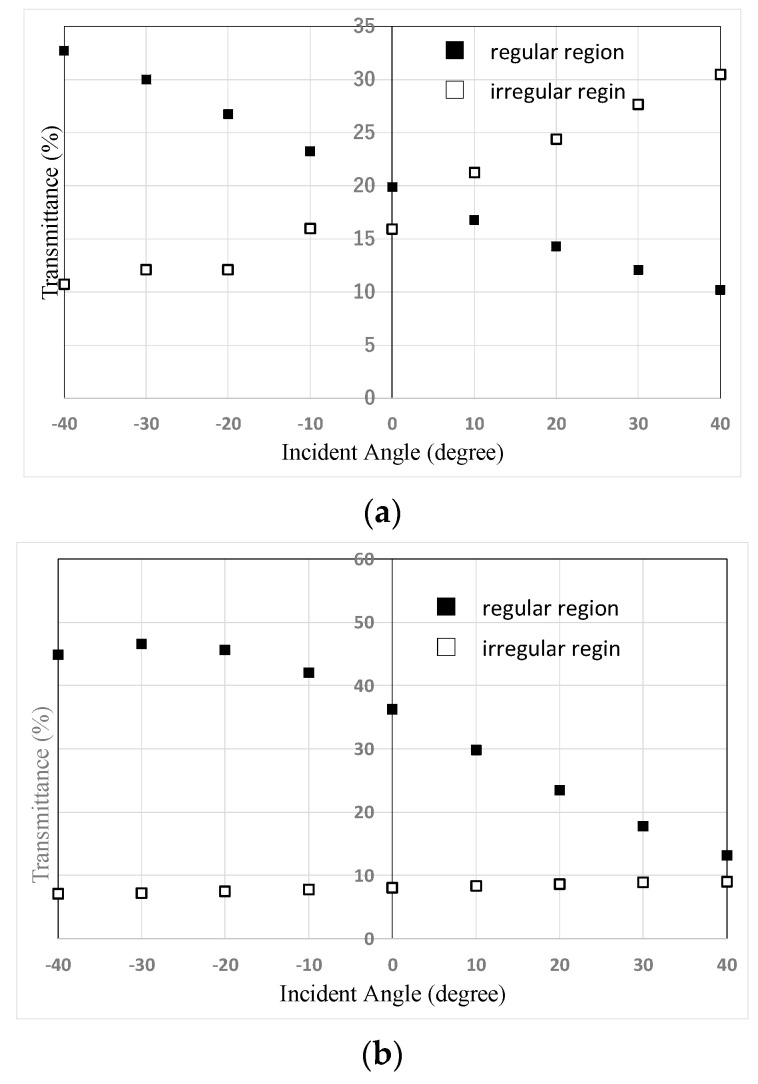
The dependence of the transmittance on the incident angle in regular and irregular regions (**a**) with a low-pretilt-angle alignment layer or (**b**) with a high-pretilt-angle alignment layer.

Opposite dependence of the transmittance on the incident angle was observed in each region (Figure 7). In the area deemed regular, the LC molecular polar angle direction was the same as the pretilt-angle direction on the alignment layer. In the area deemed irregular, the LC molecular polar angle direction was opposite to the pretilt-angle direction. However, the values of transmittance dependent on the incident angle in two regions were the same (Figure 7a). In Figure 6a, the difference between the dark area and the light area shows the transmittance contrast between the positive and negative directions shown in Figure 5.

With the use of a 25-degree pretilt-angle alignment layer, the irregular regions were reduced (Figure 6b). In Figure 7b, the dependence of the transmittance on the incident angle in the regular area was as expected for a high-pretilt-angle alignment layer [4]. However, dependence was not observed in the irregular area.

**Figure 8:**
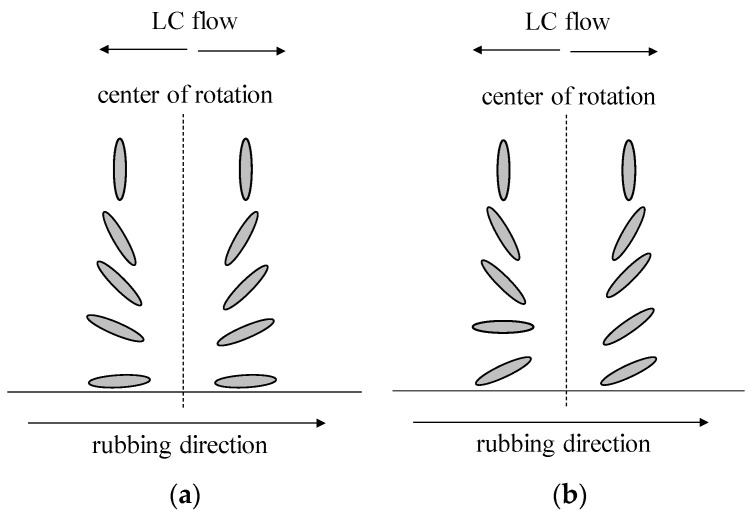
The LC molecular arrangements in a HAN-type LC film produced by spin coating with a low-pretilt-angle alignment layer (**a**) or with a high-pretilt-angle alignment layer (**b**).

During the spin-coating process, centrifugal force is applied to the solution in a liquid crystal state. In the LC layer, migration of the solution near the substrate surface is limited; however, near the interface with the air, the solution flows toward the outside of the rotation. As a result, the polar angle direction is reversed around the center of the rotation, although the alignment direction is parallel to the rubbing direction. In the case of a low pretilt angle, the effect of the pretilt angle is limited. The polar angle distribution is determined by the centrifugal force. However, in the case of a high pretilt angle, the effect of the pretilt angle increases. The expected LC molecular arrangements for each case are shown in Figure 8a,b.

### 3.2. Observed Defects in the Case of LC Layer Formation Using a Baker-Type Film Applicator

HAN-type LC film was formed using a Baker-type film applicator as detailed in Section 3.4. Some defects were observed where uniform layer formation was hindered. We call this type of defect “tilt reverse” [29,30]. In these defects, the direction of the polar angle was opposite to that in the regular area. From the direction in which light passes unhindered through the film, tilt reverse is observed as dark spots. From the opposite direction, tilt reverse is observed as bright spots in the dark area. By using a polarized microscope, the defects were observed as spots surrounded by linear defects. These defects are expected to form when irregular flow of the LC solution occurs, for example, around particles or on a deformed alignment layer surface.

### 3.3. Dependence of the Transmittance on the Incident Angle for a HAN-Type LC Layer Using Low-Pretilt-Angle Alignment Layers, Observed by Polarized Light

**Figure 9:**
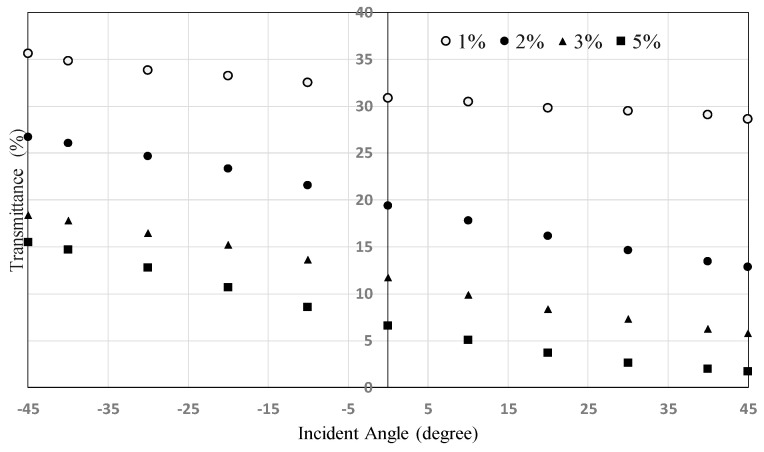
Dependence of the transmittance on the incident angle using polarized light for HAN-type LC films on glass substrate (see Figure 2 and Figure 5). The HAN-type LC films were produced by spin coating. The dependence in the regular region is shown. The light wavelength was 550 nm. The film widths were 3.6 μm (1%), 4.2 μm (2%), 3.2 μm (3%), and 3.8 μm (5%). The pretilt angle of the alignment layer was 4 degrees.

The dependence of the transmittance on the incident angle for HAN-type LC film on a glass substrate using polarized light parallel to the LC alignment direction is shown in Figure 9. Transmittance increases monotonically from +45 degrees to −45 degrees. The degree of the dependences of the transmittance on the incident angles for 2%, 3%, and 5% does not show the difference. However, the one for 1% is much smaller.

**Figure 10:**
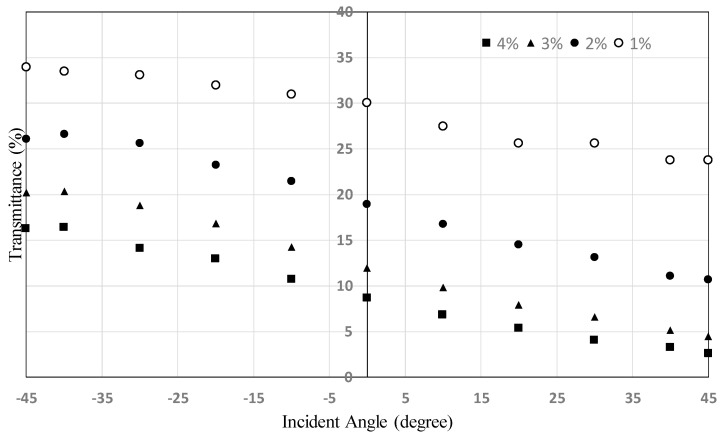
Dependence of the transmittance on the incident angle using polarized light for HAN-type LC films on PET substrate (see Figure 2 and Figure 5). The light wavelength was 550 nm. The pretilt angle of the alignment layer was 2 degrees.

Figure 10 shows the dependence of the transmittance on the incident angle using polarized light for HAN-type LC films on PET substrates. It shows a similar tendency to that indicated in Figure 9. The dependence can be explained by the mechanism shown in Figure 2. Incident light from the + direction shown in Figure 5 proceeds in the direction perpendicular to the dichroic dye molecular axis and is efficiently absorbed by the dye. On the other hand, incident light from the − direction proceeds parallel to the dye molecular axis, and the absorption is therefore limited. The slopes of the graphs for the concentrations from 2% to 5% do not change, but that for 1% decreases considerably.

**Figure 11:**
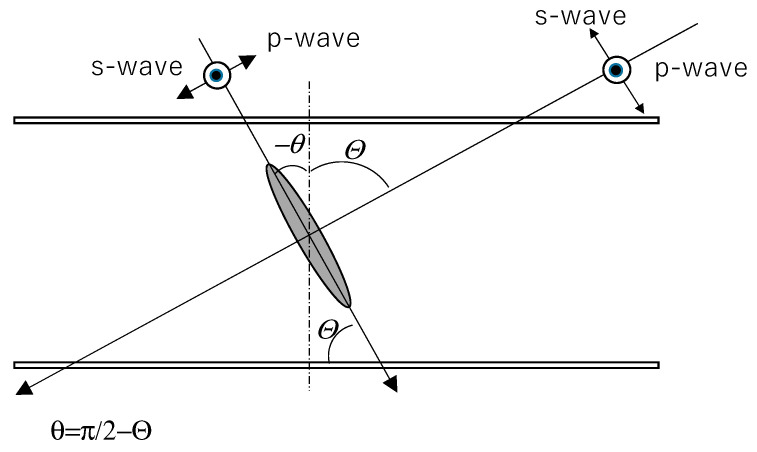
An LCD structure in which the polar angle of the LC molecule is a constant value of *θ*.

To explain the mechanism, the LCD structure shown in Figure 11 is considered. For simplicity, the polar angle of the LC molecule is assumed to be 45 degrees. Incident angles of 45 degrees and −45 degrees are considered. By the Lambert–Beer law, the transmittance at incident angles of +45 degrees and −45 degrees for p-waves *T*_+45_ and *T*_−45_ can be expressed by the equations below.

$$T_{+45}=10^{-\varepsilon_{s}lc}$$

$$T_{-45}=10^{-\varepsilon_{p}lc}$$

The passing distances *l*_+45_ and *l*_−45_ for both incident angles are the same value, *l*. $\varepsilon_{p}$ and $\varepsilon_{s}$ are the absorption coefficients of polarized light vibrating parallel and perpendicular to the molecular long axis of the dye. The dependence of the transmittance on the incident angle can then be estimated as the value *T*_−45_ − *T*_+45_:

$$T_{-45}-T_{+45}=T_{-45}\left\{1-10^{-\left(\varepsilon_{p}-\varepsilon_{s}\right)lc}\right\}$$

The equation above shows that the dependence of the transmittance on the incident angle depends on the film thickness and on the concentration and $\varepsilon_{p}-\varepsilon_{s}$ value of the dye. It also shows that in the case of a high concentration or thick film, the change in the dependence due to change in the concentration or the thickness becomes small. In Figure 9 and Figure 10, the change in the dependence from 1% to 2% is larger than that from 2% to 5%. To obtain high dependence, the use of a high concentration of the dye and a thick film would be effective. However, these values cannot be changed freely, because the transmittance varies depending on these values. The most effective way to obtain high dependence would then be the use of dye possessing a large $\varepsilon_{p}-\varepsilon_{s}$ value.

Furthermore, the degree of alignment or order parameter of the LC material is important. The degree of liquid crystal alignment depends on the LC material, the alignment layer, and the process of film formation. These factors can improve the dependence of the transmittance on the incident angle.

### 3.4. Relationship between the Alignment Layer Pretilt Angle and the Dependence of the Transmittance on the Incident Angle

**Figure 12:**
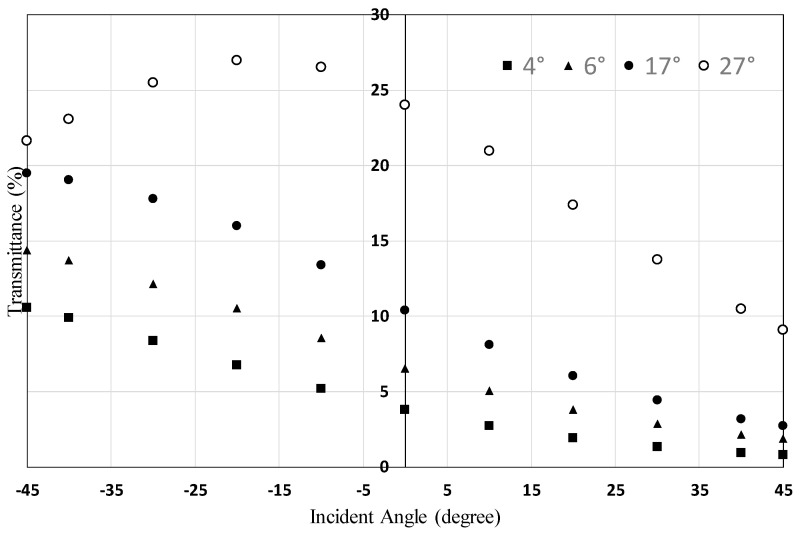
Dependence of the transmittance of HAN-type LC films, made using alignment layers with different pretilt angles, on the incident angle of polarized light (see Figure 2 and Figure 5). The concentration of the dichroic dye was 5%.

Figure 12 shows the dependence of the transmittance of HAN-type LC films, made using alignment layers with different pretilt angles, on the incident angle of polarized light. For alignment layers with pretilt angles of 4, 6, and 17 degrees, the transmittance increases from 45 degrees to −45 degrees monotonically. The transmittance at 45 degrees takes almost the same value for these three films. However, as the pretilt angle increases, the dependence of the transmittance on the incident angle increases. On the other hand, in the case of a pretilt angle of 27 degrees, the transmittance peaks at −20 degrees. Figure 12 shows that a 17-degree pretilt angle is preferable for HAN-type LC films.

### 3.5. Combination of Two HAN-Type LC Films and a Half-Wave Plate

**Figure 13:**
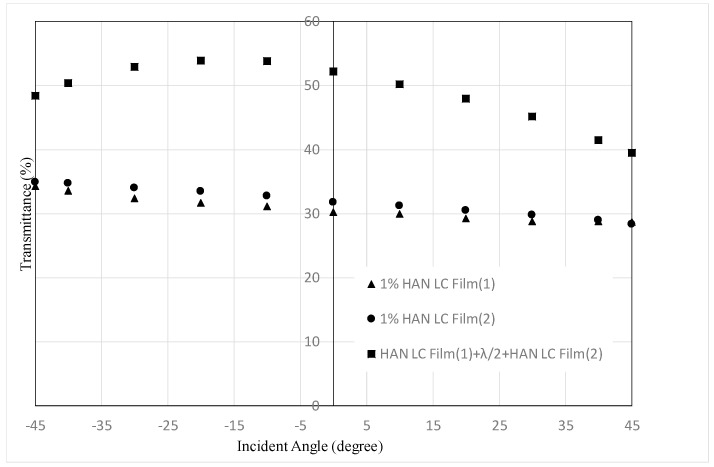
Dependence of the transmittance on the incident angle for a combination of two HAN-type LC films on glass substrates and a half-wave plate, shown in Figure 3, and that for a single HAN-type LC film with a polarizer. The concentration of the dichroic dye was 1%.

**Figure 14:**
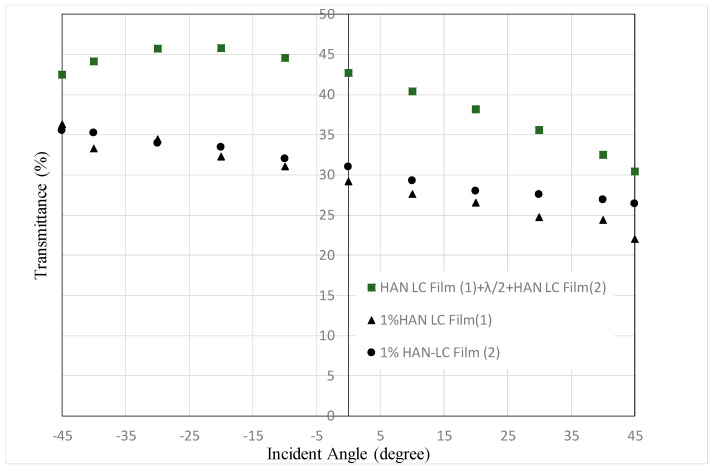
Dependence of the transmittance on the incident angle for a combination of two HAN-type LC films on PET substrates and a half-wave plate, shown in Figure 3, and that for a single HAN-type LC film with polarizer. The concentration of the dichroic dye was 1%.

Figure 13 and Figure 14 show the transmittance dependences on the incident angle for the combination of two HAN-type LC films (1% of dye concentration) and a half-wave plate shown in Figure 3, alongside that for single HAN-type LC film (1% of dye concentration) with a polarizer. The transmittances for negative incident angles are more than 50% and 43%, respectively. These values cannot be achieved by the filters with a polarizer. In Figure 13, at +45° the transmittance of two LC films and a half-wave plate is much higher than the one of an LC film with a polarizer, because p-wave from the direction of +*θ* cannot be absorbed sufficiently due to the low dye concentration and thin film thickness compared with a polarizer.

In Figure 14, the transmittance of two LC films and a half-wave plate is similar to those for the one of an LC film with a polarizer. It shows the result that can be expected by the mechanism shown in Figure 4. In both Figure 13 and Figure 14, the maximum values can be observed around −20°. This phenomenon could be observed for the devices using LCDs [4]. With a large incident angle, the transmittance could decrease as the optical path extends. As a result, the maximum transmittance could be observed at an incident angle smaller than 45 degrees. In the case of two LC films and a half-wave plate, the maximum values could be observed at smaller incident angles than that for an LC film with a polarizer, because the optical path doubles.

**Figure 15:**
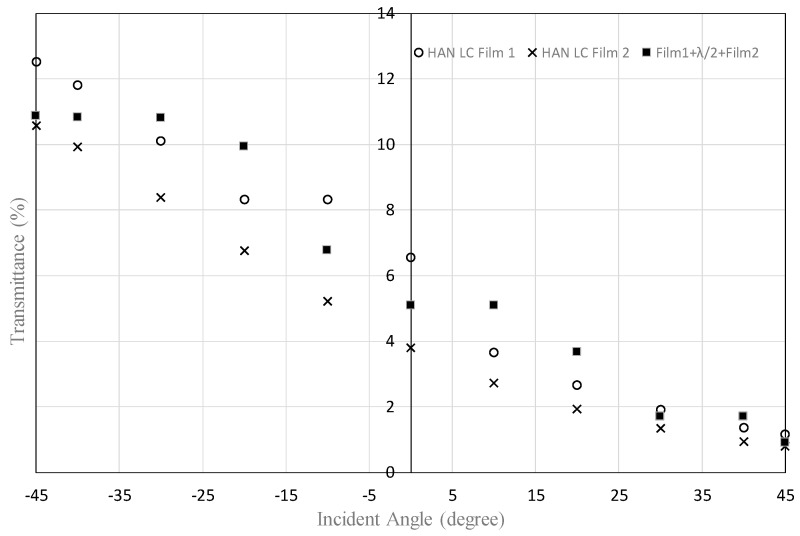
Dependence of the transmittance on the incident angle for a combination of two HAN-type LC films on glass substrates and a half-wave plate, shown in Figure 3, and that for a single HAN-type LC film with a polarizer. The concentration of the dichroic dye was 5%.

**Figure 16:**
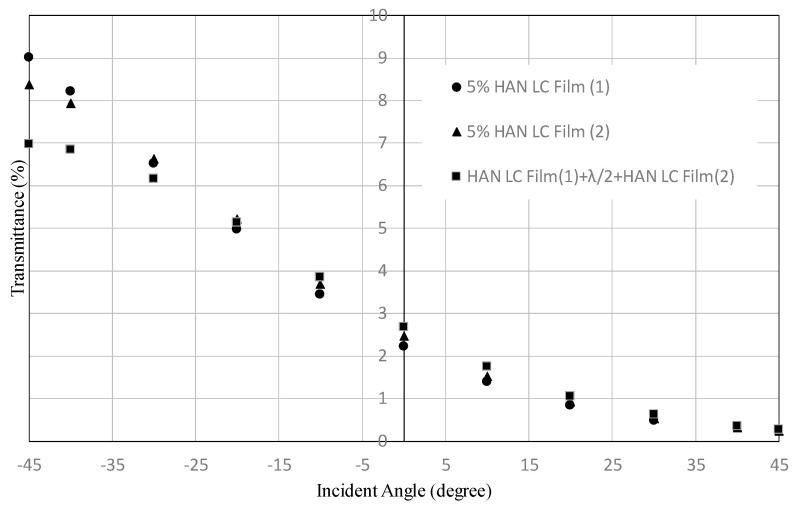
Dependence of the transmittance on the incident angle for a combination of two HAN-type LC films on PET substrates and a half-wave plate, shown in Figure 3, and that for a single HAN-type LC film with polarizer. The concentration of the dichroic dye was 5%.

Figure 15 and Figure 16 show the transmittance dependences on the incident angle for the filters using HAN-type LC films (5% of dye concentration). In both figures, in the region of positive incident angle, the transmittances of the two LC films with a half-wave plate are the same as those for an LC film with a polarizer. This result can be expected by the mechanism shown in Figure 4. However, in the region of negative incident angle, the transmittances of the two LC films with a half-wave plate are similar to the ones for an LC film with a polarizer. The result cannot be explained by the mechanism shown in Figure 4. This could be explained by two factors: the order parameter of the LC film is not enough and the polar angle is distributed from 0° to 90° for HAN-LC film. In the case of high concentration of the dichroic dye, the incident light from −45° would be absorbed by the dyes at the distributed polar and azimuth angles. To realize a function of two LC films with a half-wave plate, as shown in Figure 4, the appropriate concentration of the dichroic dye should be important.

A wide variety of applications can be expected for these filters. For architectural window applications, the preferable transmittance could be varied depending on the area or the purpose of the usage. When the high transmittance is preferable, the filter with two LC films and a half-wave plate could be selected. For car side windows and sunglasses, relatively low transmittance would be required. For these products, the filter of one LC film with a polarizer could be selected.

### 3.6. Applications

**Figure 17:**
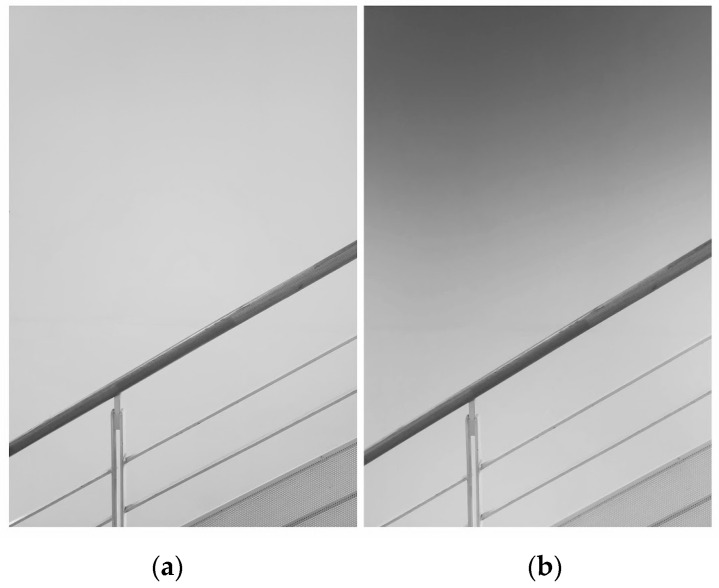
Photographs of a white wall without a louver LC filter (**a**) and with a louver LC filter (**b**). The louver LC filter was composed of HAN-type LC film and a polarizer.

**Figure 18:**
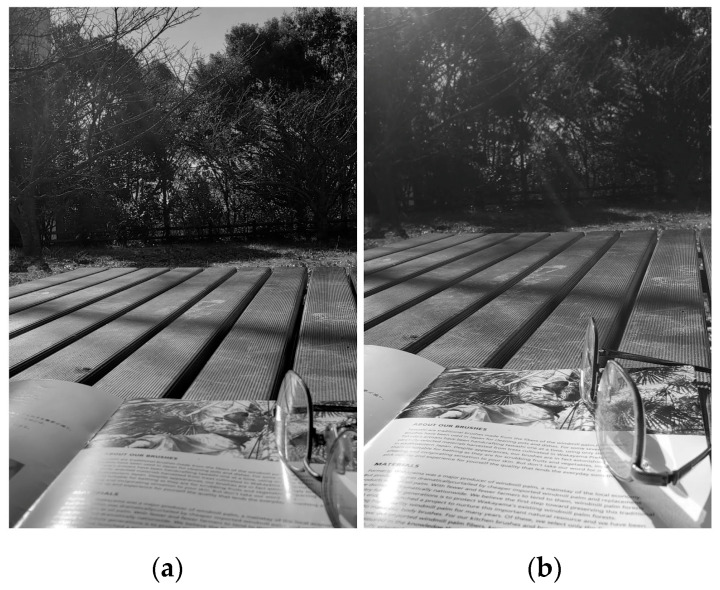
Photographs of scenery and a book without a louver LC filter (**a**) and with a louver LC filter (**b**).

**Figure 19:**
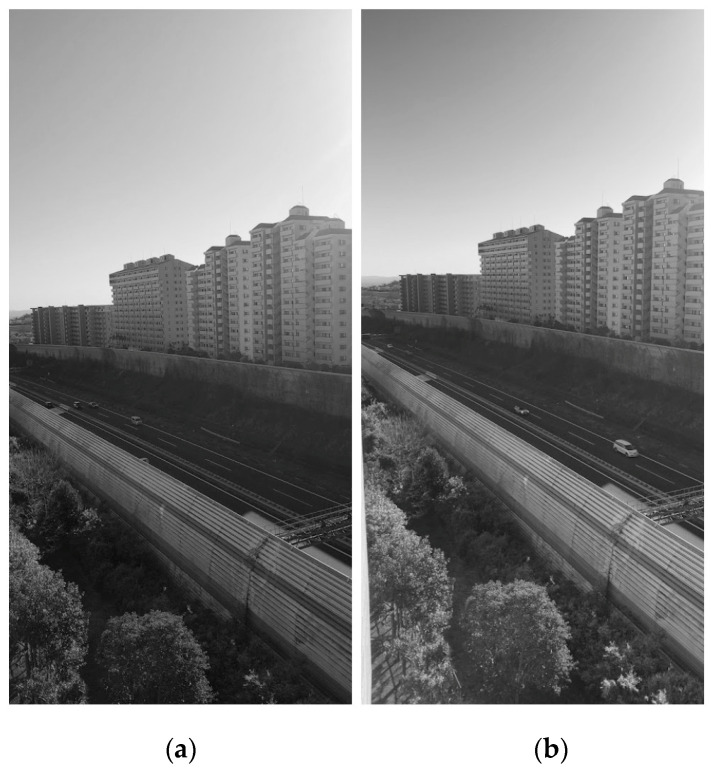
Photographs of a view from a high building without a louver LC filter (**a**) and with a louver LC filter (**b**).

Louver LC filters are optical filters through which the transmittance varies depending on the incident angle. With this property, the louver LC filters could be applied to sunglasses and windows, among others. Figure 17, Figure 18 and Figure 19 show photographs taken with and without a louver LC filter. The louver LC filter was composed of HAN-type LC film and a polarizer, as shown in Figure 2. With a louver LC filter, the total light strength decreases. However, the photograph brightness is maintained automatically. Figure 17b clearly shows that the strength of the light decreases only from the upper side. In Figure 18 and Figure 19, with a louver LC filter, the lower parts of the photographs look brighter. Figure 18 shows that by looking through the louver LC filter, we could read the documents more clearly than we could by using conventional sunglasses. In Figure 17, the scenery in the lower part can be observed more brightly and precisely than it can without a louver LC filter.

## 4. Conclusions

In this article, optical filters with transmittance dependent on the incident angle, made using liquid crystalline ink, are proposed. We refer to this optical filter as a “louver LC filter”. For louver LC filters, HAN-type LC films produced from an LC monomer and dichroic dye were used. Two kinds of louver filters are proposed: one is composed of a HAN-type LC film and a polarizer, and the other is composed of two HAN-type LC films with a half-wave plate between them. By using these louver LC filters, the light strength from the upper side can be reduced preferentially. This property is appropriate for sunglasses, windows in cars and buildings, and other applications.

## 5. Patents

Patent WO2021256499A1, Optical element and eyewear, has been filed.
